# Initiation of L-DOPA Treatment After Detection of Diabetes-Induced Retinal Dysfunction Reverses Retinopathy and Provides Neuroprotection in Rats

**DOI:** 10.1167/tvst.10.4.8

**Published:** 2021-04-13

**Authors:** Kyle Chesler, Cara Motz, Harrison Vo, Amber Douglass, Rachael S. Allen, Andrew J. Feola, Machelle T. Pardue

**Affiliations:** 1 Atlanta VA Healthcare System, Atlanta, GA, USA; 2 Biomedical Engineering, Georgia Institute of Technology and Emory University, Atlanta, GA, USA

**Keywords:** diabetes, retina, dopamine, retinopathy, neuroprotection

## Abstract

**Purpose:**

L-DOPA treatment initiated at the start of hyperglycemia preserves retinal and visual function in diabetic rats. Here, we investigated a more clinically relevant treatment strategy in which retinal and visual dysfunction designated the beginning of the therapeutic window for L-DOPA treatment.

**Methods:**

Spatial frequency thresholds using optomotor response and oscillatory potential (OP) delays using electroretinograms were compared at baseline, 3, 6, and 10 weeks after streptozotocin (STZ) between diabetic and control rats. L-DOPA/carbidopa treatment (DOPA) or vehicle was delivered orally 5 days per week beginning at 3 weeks after STZ, when significant retinal and visual deficits were measured. At 10 weeks after STZ, retinas were collected to measure L-DOPA, dopamine, and 3,4-dihydroxyphenylacetic acid (DOPAC) levels using high-performance liquid chromatography.

**Results:**

Spatial frequency thresholds decreased at 6 weeks in diabetic vehicle rats (28%), whereas diabetic DOPA rats had stable thresholds (<1%) that maintained to 10 weeks, creating significantly higher thresholds compared with diabetic vehicle rats (*P* < 0.0001). OP2 implicit times in response to dim, rod-driven stimuli were decreased in diabetic compared with control rats (3 weeks, *P* < 0.0001; 10 weeks, *P* < 0.01). With L-DOPA treatment, OP2 implicit times recovered in diabetic rats to be indistinguishable from control rats by 10 weeks after STZ. Rats treated with L-DOPA showed significantly increased retinal L-DOPA (*P* < 0.001) and dopamine levels (*P* < 0.05).

**Conclusions:**

L-DOPA treatment started after the detection of retinal and visual dysfunction showed protective effects in diabetic rats.

**Translational Relevance:**

Early retinal functional deficits induced by diabetes can be used to identify an earlier therapeutic window for L-DOPA treatment which protects from further vision loss and restores retinal function.

## Introduction

Diabetes mellitus (DM) is a major public health problem across the globe that is approaching epidemic proportions.^1^ Diabetic retinopathy (DR) is the most common complication of diabetes.^2^ With DM rapidly increasing in global prevalence, DR cases are expected to double from 7.7 million to 14.6 million people by 2050 in the United States.^3^

In the clinic, vascular abnormalities observed in a patient's fundus photograph are used to diagnose DR. Although there is no cure for DR, current therapeutic strategies are aimed at preventing or delaying progression to the late stages of the disease. These interventions, such as panretinal photocoagulation and intravitreal injections of anti–vascular endothelial growth factor (VEGF) agents, are invasive and expensive, and aim to slow the progression of proliferative, vision-compromising DR. Despite these treatment options, DR remains the leading cause of blindness in working age adults in the United States,^1^ demonstrating the need for earlier detection and treatment.

Increasing evidence suggests that DR can be detected and even treated before clinically diagnosed fundus defects using electroretinograms (ERG).^4^^,^^5^ Since the 1960s,^6^ decreased and delayed oscillatory potentials (OPs) in patients with DM or animal models of DM have been consistently observed using ERG before vascular pathologies, such as pericyte dropout, are present.^7^^–^^15^ Diabetes-induced changes in the ERG may be attributed to neuronal dysfunction,^7^^,^^11^^,^^16^^,^^17^ changes in the levels of neurotransmitters,^17^^–^^20^ and cell death in the neural retina.^18^ In addition, early vascular changes, such as increased vascular permeability as early as 1 week after hyperglycemia in diabetic rodents,^21^^,^^22^ or structural vascular abnormalities detected with optical coherence tomography angiography in patients with diabetes and no visible microvascular fundus changes^23^^–^^25^ may alter neuronal function owing to neurovascular coupling.

In addition to ERG changes, animal models and patients with DM exhibit subtle visual defects before the clinically recognized vascular defects in DR appear.^26^^,^^27^ An effective behavioral test of visual function in rats is assessment of the optomotor response (OMR), which uses an animal's head turn response to measure its ability to track moving stimuli.^28^^,^^29^ The OMR has been used to detect decreased spatial frequency (SF) thresholds in streptozotocin (STZ)-induced DM rats that model early visual defects observed in patients with DR.^17^^,^^27^

Although the etiology of DR is poorly understood, there is evidence that dopamine deficiencies in the diabetic retina contribute to preclinical retinal defects of the diabetic retina.^5^^,^^17^^,^^30^ Furthermore, dopaminergic amacrine cell loss has been shown to occur in rat models of DR.^18^ Dopamine is a crucial neurotransmitter in both the brain and retina. The precursor to dopamine, l-3,4-dihydroxyphenylalanine, or L-DOPA, is used as a treatment to increase endogenous dopamine levels. L-DOPA, unlike dopamine, can cross the blood–brain barrier, where it is taken up by cells and converted into dopamine.^31^ L-DOPA treatment has been shown to be neuroprotective against the early retinal dysfunction associated with DR in both diabetic humans and animals.^5^^,^^17^^,^^30^^,^^32^

Diabetic rodents treated with L-DOPA at the onset of diabetes show preserved retinal and visual function.^17^^,^^27^^,^^30^ In these preclinical rodent studies, L-DOPA treatment was initiated immediately after the onset of diabetes and offer limited clinical translatability. However, L-DOPA could be prescribed after the first signs of early stage DR as detected by retinal or visual dysfunction. The goals of this study were (1) to evaluate the efficacy of a clinically relevant dosing paradigm that begins L-DOPA treatment after detection of visual and retinal defects, and (2) to assess whether L-DOPA treatment can recover retinal dopamine levels in diabetic rats.

## Methods

### Animals

Male Long Evans rats were obtained from Charles Rivers Laboratories (Wilmington, MA) and housed in the animal facility at the Atlanta Veterans Affairs Healthcare System (Decatur, GA) under a 12:12-hour (light:dark) cycle with food and water ad libitum. All procedures were approved by the Institutional Animal Care and Use Committee of the Atlanta Veterans Affairs Healthcare System and performed in full accordance with the ARVO Statement for the Use of Animals in Ophthalmic and Vision Research.

### Experimental Design

**Figure 1.**
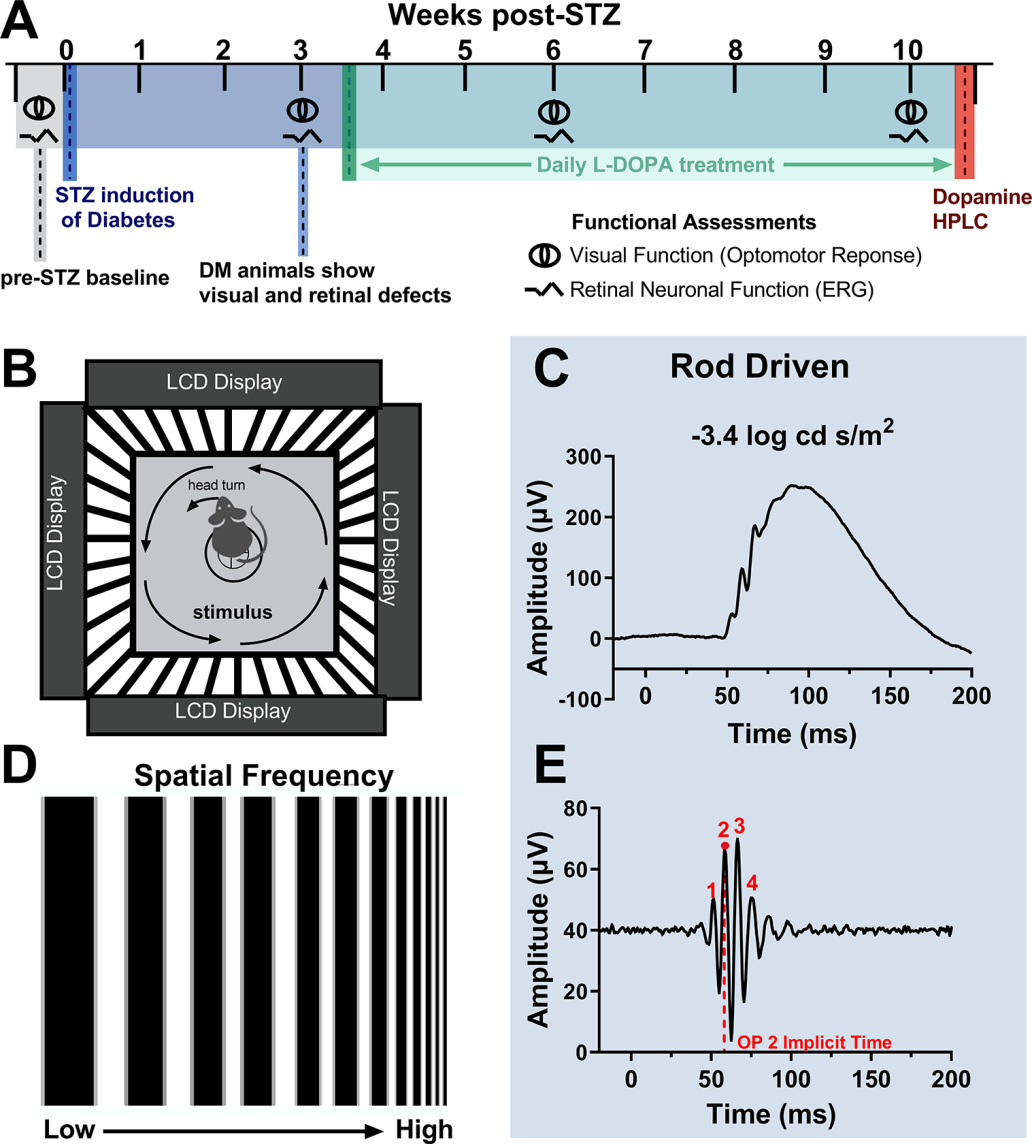
Experimental design and methodology. (A) Baseline functional assessments are taken at week 0 before STZ induction of diabetes (DM). STZ induction of DM is indicated by the dark blue line. Visual and retinal functional assessments are taken again at 3 weeks after STZ, before initiating the daily L-DOPA treatment which is indicated by the green line. L-DOPA treatment continues throughout the green shaded region. Visual and retinal functional assessments are repeated at 6 and 10 weeks after STZ. After 10 weeks after STZ, animals are euthanized and retinas are collected for HPLC to measure retinal L-DOPA, dopamine, and DOPAC levels, indicated by the red line. (B) A cartoon of a rat undergoing OMR assessment of visual function while sitting on a pedestal surrounded by four LCD monitors to create a virtual drum. The stimulus changes in SF are described in (D). (C, E) ERG recordings and corresponding filtered OP tracings for dim, rod-driven flash stimuli. Red numbers indicate the OP peaks and the red dotted line represents OP2 implicit time.

Baseline measurements of retinal and visual function were taken at week zero before the administration of STZ. Diabetes was induced at week zero. At 3 weeks after STZ, retinal and visual function were measured via ERG and OMR, respectively. After confirmation of retinal and visual deficits at 3 weeks after STZ, daily (5 days/week) L-DOPA treatment began. At 6 and 10 weeks after STZ, retinal and visual function were measured again. At 10 weeks after STZ, rats were euthanized via decapitation, and the retinas were extracted for L-DOPA, dopamine, and 3,4-dihydroxyphenylacetic acid (DOPAC) analysis via high-performance liquid chromatography (HPLC). Retinas were flash-frozen in liquid nitrogen and extracted between the hours of 10:00 AM and 12:00 PM. The experimental timeline is shown in Figure 1A.

### Oral L-DOPA Treatment

Rats were trained to consume controlled doses of L-DOPA suspended in sweetened, water-based vehicle from a syringe (Chesler et al, unpublished data, July 2019). Once trained, L-DOPA–treated rats consumed 20 mg/kg L-DOPA/5 mg/kg carbidopa voluntarily from a syringe. The L-DOPA was prepared with 10 mg/mL L-DOPA (Sigma-Aldrich, St. Louis, MO) and 2.5 mg/mL carbidopa monohydrate (Abcam, Cambridge, MA) to emulate a commonly prescribed drug combination for Parkinson's disease.^33^ Carbidopa is a peripheral decarboxylase inhibitor that minimizes the amount of L-DOPA that oxidizes to dopamine in the periphery to maximize the amount that can pass the blood–brain barrier and act on retinal and neuronal tissue. Once combined, the solution was vortexed thoroughly in a sweetened, water-based vehicle solution (MediDrop Sucralose; Clear H2O, Portland, ME) to create a homogenous suspension of L-DOPA/carbidopa. The L-DOPA/carbidopa cocktail was made fresh weekly, stored in a refrigerated, light-proof container, thoroughly remixed before use and kept cold during use. We were cautious to select a dosage that was lower than the typical dosage used to induce dyskinesia (25 mg/kg)^34^ yet could still increase retinal dopamine levels.^35^

### Hyperglycemia Induction

Hyperglycemia was induced with a single intravenous injection of STZ (100 mg/kg; Sigma-Aldrich) dissolved in citrate buffer (pH 4.0). Control rats were injected with vehicle alone. Diabetes was defined as two successive daily blood glucose levels higher than 250 mg/dL (freestyle handheld blood glucose meter from tail-prick blood), which routinely occurred 2 to 3 days after STZ injection. Body weights and blood glucose were monitored two to three times per week. DM rats were treated with small pellets of sustained-release subcutaneous insulin (Linplant; Linshin Canada, Scarborough, ON, Canada) at a dose sufficient to prevent excessive weight loss and catabolic response, but insufficient to control hyperglycemia.^36^

### Visual Function: SF Thresholds with OMR

The visual function of each rat, without movement restriction, was tested using the virtual optokinetic system (OptoMotry system; Cerebral-Mechanics, Lethbridge, AB, Canada) as described elsewhere.^28^ Briefly, the rat was placed on a platform at the center of a virtual reality chamber composed of four computer monitors that display vertical sine wave gratings rotating at a speed of 12 deg/s. The experimenter monitored the rat in real time through a video camera positioned above the animal and noted the presence or absence of reflexive head movements (tracking) in response to the rotating gratings in the same direction. This set up is described in Figure 1B. The experimenter also manually tracked the head of the rat to align the center of the virtual cylinder to the viewing position of the rat. To determine the SF threshold, the grating started at a SF of 0.042 cyc/deg with 100% contrast. The SF threshold was determined automatically by the OMR software using a staircase paradigm (Figs. 1B and 1D).

### Retinal Function: Electroretinography

Rats were dark adapted for 3 hours and then prepared under dim red illumination as previously described.^27^ In brief, rats were anesthetized (ketamine [60 mg/kg] and xylazine [7.5 mg/kg]), pupils dilated (1% tropicamide), and the corneal surface anesthetized (0.5% tetracaine HCl). Using a custom made gold loop electrode, responses were recorded to flash stimuli presented in order of increasing luminance using a signal-averaging system (UTAS BigShot; LKC Technologies, Gaithersburg, MD). ERG stimuli consisted of a five-step dark-adapted series at –3.4, –1.5, –0.6, 1.5, and 1.9 log cd s/m^2^, followed by a one-step light-adapted flicker at 2 log cd s/m^2^. Animals were light adapted for 10 minutes before exposure to the flicker stimulus. Only the 3.4, –0.6, and 1.9 log cd s/m^2^ dark-adapted ERGs are included here. ERGs were done 30 minutes to 1 hour after any treatment given (vehicle or L-DOPA). After testing, rats received atipamezole hydrochloride (1 mg/kg; Antisedan, Pfizer Animal Health, New York, NY) to reverse the effects of the xylazine. The OPs were filtered digitally using custom MATLAB software (Mathworks, Cambridge, MA) (75–350 Hz). For each animal, the ERG waveform with the greatest amplitudes was used as the representative value for that animal. ERG a- and b-wave amplitudes and implicit times were measured, as reported elsewhere.^17^^,^^27^ The implicit times of individual OP1 through OP4 were determined and OP2 results are reported here. Figures 1C and 1E demonstrate how the OP2 implicit time was identified for each flash intensity.

### HPLC Analysis of L-DOPA, Dopamine, and DOPAC Content

Frozen retinas were homogenized in 0.4 N perchloric acid to extract monoamines and then centrifuged at 10,000×*g*. Supernatant was collected for HPLC assessment of L-DOPA, dopamine, and DOPAC, and the remaining protein pellets were processed using the Pierce BCA assay (ThermoFisher Scientific, Waltham, MA) for total protein quantifications. Monoamine levels in the retina were normalized to total protein concentration of tissue. Monoamines were examined by HPLC with electrochemical detection as described previously.^37^ For HPLC, an ESA 5600A CoulArray detection system (Bedford, MA), equipped with an ESA Model 584 pump and an ESA 542 refrigerated autosampler was used. Separations were performed using an MD-150 × 3.2 mm C18 (3 µM) column at 23°C. The mobile phase consisted of 8% acetonitrile, 75 mM NaH_2_PO4, 1.7 mM 1-octanesulfonic acid sodium, and 0.025% trimethylamine at a pH of 3.0. A 25-µL of sample was injected. The samples were eluted isocratically at 0.4 mL/min and detected using a 6210 electrochemical cell (ESA) equipped with 5020 guard cell. The guard cell potential was set at 500 mV, while analytical cell potentials were −175, 150, 350, and 425 mV. The analytes were identified by the matching criteria of retention time to known standards (Sigma Chemical Co., St. Louis, MO) of L-DOPA, dopamine and DOPAC. Compounds were quantified by comparing peak areas with those of standards on the dominant sensor. The monoamine content was then normalized to total protein quantifications for each retina to account for variability in the sizes of retinas collected from different animals.

### Statistical Analysis

Statistical analysis was performed using statistical software (GraphPad Prism version 8.0.0 for Mac, GraphPad Software, San Diego, CA; available: www.graphpad.com). The 3-week visual acuity assessment was statistically analyzed using a two-tailed Student's t-test. All other statistics reported are two- or three-way analysis of variance (ANOVA) interaction effects unless otherwise stated, followed by Holm-Sidak or Tukey's tests for individual comparisons where applicable. Statistical significance threshold was such that *P < 0.05; **P < 0.01; ***P < 0.001, and ****P < 0.0001.

## Results

### At 3 Weeks After STZ, Diabetic Animals Showed Significant Visual and Retinal Defects

**Figure 2.**
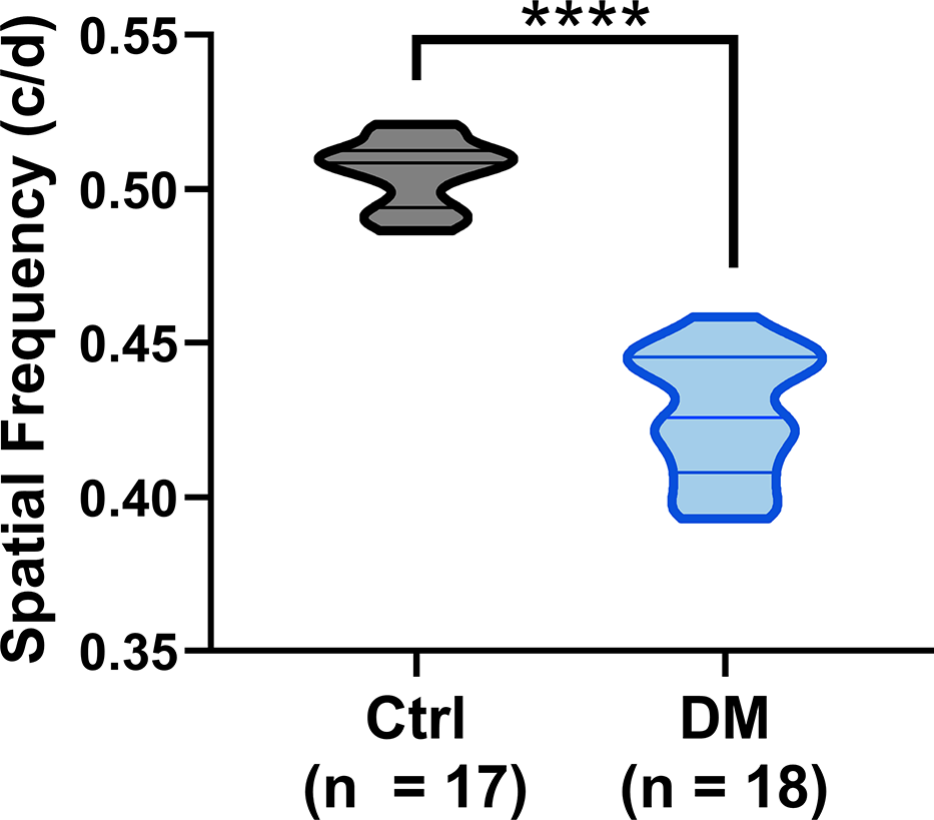
Diabetic rats show significant visual deficits at 3 weeks after STZ. STZ-induced DM rats showed significantly reduced SF thresholds compared with control rats (Student *t* test, ***P < 0.0001). The violin plot shows the distribution of the data points with median and quartiles indicated by the middle and top/bottom lines, respectively.

To assess the effects of sustained hyperglycemia on visual function at 3 weeks after STZ, we measured SF thresholds using OMR. Although the control and DM groups had similar SF thresholds at baseline to those reported previously in Long Evans rats,^28^ DM rats had a significant 16.5% decrease in their visual acuity at 3 weeks after STZ compared with control rats (Fig. 2; Student *t* test = 14.01, df = 33, *P* < 0.0001).

**Figure 3.**
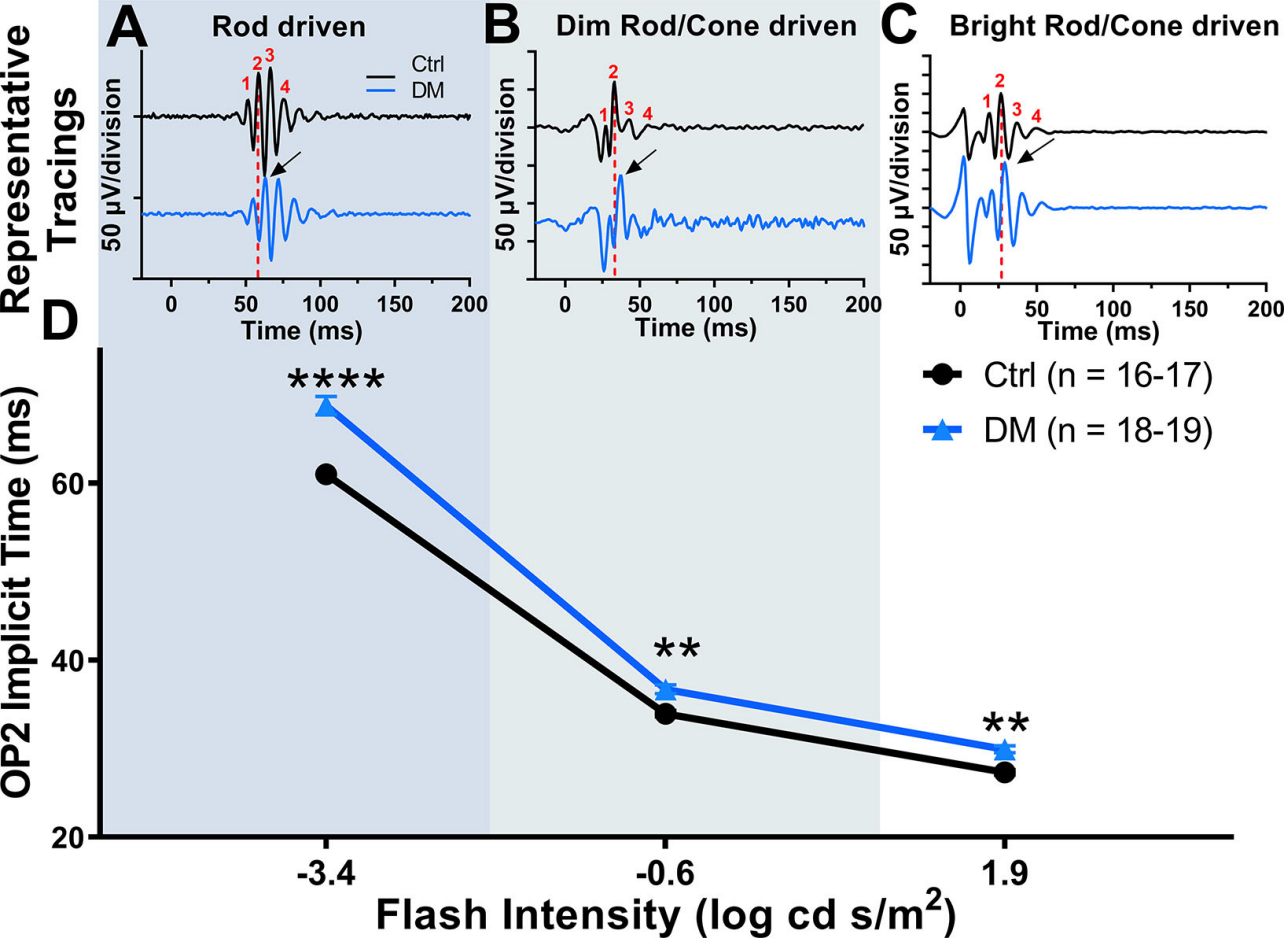
Diabetic rats show significantly delayed OP2 implicit times at 3 weeks after STZ. (A, B, C) Representative OP tracings for control and diabetic animals at the 3-week time point for each flash condition. The red dotted line represents OP2 implicit time for the representative control tracing, demonstrating the delay in OP2 implicit time of DM rats (black arrow). (D) OP2 implicit times plotted for three flash stimuli that stimulate rod-dominated, and rod/cone driven responses show significant delays, two-way repeated-measure ANOVA, main effect of diabetes, F (2, 54) = 7.802, *P* = 0.0011. Data plotted as mean with standard error of the mean. **P < 0.01; ****P < 0.0001.

We then assessed retinal function across rod-dominated and mixed rod/cone ERG responses at 3 weeks after STZ. Similar to previous findings,^17^^,^^38^ diabetic rats showed significant OP delays at 3 weeks after STZ across all flash intensities (Fig. 3D), two-way repeated-measures ANOVA interaction effect, F (2, 54) = 7.802; *P* = 0.0011. Rod-driven ERGs in response to dim flash stimuli (–3.4 log cd s/m^2^) showed the most significant delays in OP2 of 12.6% compared with control rats (Figs. 3A and 3D; two-way repeated-measures ANOVA, Holm–Sidak post hoc, *P* < 0.0001). Dim mixed rod/cone driven OP2 responses (–0.6 log cd s/m^2^) were significantly delayed by 8.0% at 3 weeks after STZ compared with control rats (Figs. 3B and 3D; two-way repeated-measure ANOVA, Holm-Sidak post-hoc, *P* = 0.0052). Bright flash, mixed rod/cone OP2 responses (1.9 log cd s/m^2^) were also significantly delayed by 9.5% at 3 weeks after STZ compared with control rats (Figs. 3C and 3D; two-way repeated-measures ANOVA, Holm–Sidak post hoc, *P* = 0.0052).

ERG a-wave amplitudes were not different between DM and control rats. For the a-wave implicit times, a significant interaction between diabetes and L-DOPA treatment was not present, three-way mixed ANOVA, three-way interaction F (1, 19) = 1.253, *P* = 0.279. However, the a-wave implicit times were delayed in DM rats by 10 weeks after STZ, Flash × Diabetes interaction, F (1, 19) = 6.96, *P* = 0.016, and L-DOPA provided significant preservation in response to the dim mixed rod/cone–driven response (–0.6 log cd s/m^2^, *P* < 0.01), but not the bright mixed rod/cone stimuli, 1.9 log cd s/m^2^; Supplemental Figure, Flash × Treatment interaction, F (1, 19) = 8.48, *P* = 0.009. Interestingly, the diabetic groups showed significantly decreased b-wave amplitudes at 1.9 log cd s/m^2^ that did not benefit from L-DOPA treatment, three-way mixed ANOVA F (2, 112) = 0.175, *P* = 0.840; Flash × Diabetes interaction, F (2, 112) = 5.86, *P* = 0.004. Furthermore, the diabetic groups also had delayed b-wave implicit times across all flash stimuli compared with control groups, Flash × Diabetes interaction F (2, 112) = 25.21, *P* < 0.0001, and showed no benefit from L-DOPA treatments, three-way mixed ANOVA, three-way interaction F (2, 112) = 3.05, *P* = 0.051; Supplemental Figure.

### Daily L-DOPA Treatment Protected From Further Decreases in Visual Function in Diabetic Rats

**Figure 4.**
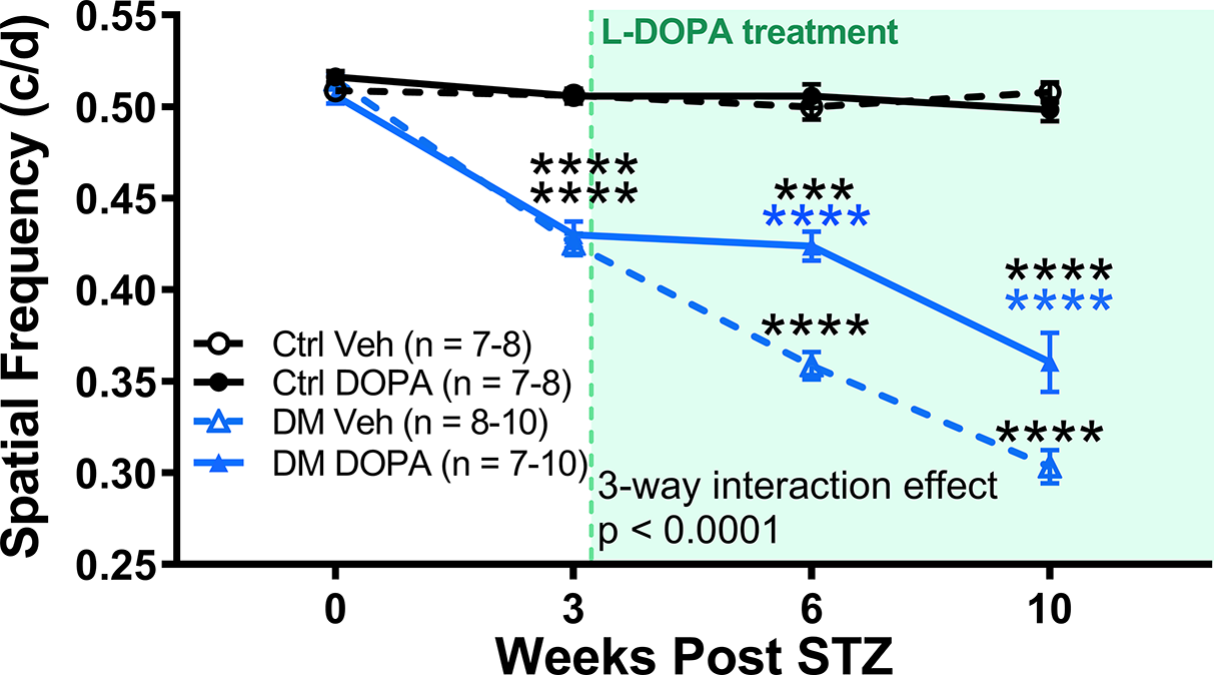
L-DOPA treatment protects visual function in diabetic rats. At the 3-week time point, DM rats showed significant reductions in SF thresholds (three-way repeated ANOVA interaction effect, (3, 85) = 10.72, *P* < 0.0001; Holm-Sidak post hoc, *P* < 0.0001). DM DOPA rats showed significant protection against further losses in SF thresholds compared with DM vehicle rats at both 6 and 10 weeks (Holm–Sidak post hoc, *P* < 0.0001). The green dotted line represents the start of daily L-DOPA treatment for DOPA rats, and the shaded green region indicates daily L-DOPA treatment for rats in DM DOPA and control DOPA groups. ***P < 0.001; ****P < 0.0001

Daily L-DOPA treatment slowed the decrease in SF thresholds in DM rats from 3 to 10 weeks after STZ (Fig. 4), three-way repeated-measure ANOVA, three-way interaction effect, F (3, 85) = 10.72, *P* < 0.0001. The SF thresholds were significantly decreased by 16.5% at 3 weeks after STZ in DM rats compared with control vehicle rats (Fig. 4). After starting L-DOPA treatment at 3 weeks after STZ, the SF thresholds of DM vehicle rats continued to significantly decrease from 16.5% at 3 weeks after STZ (Holm–Sidak post hoc, *P* < 0.0001) to 28.4% at 6 weeks after STZ (Holm–Sidak post hoc, *P* < 0.0001) compared with control vehicle rats. In contrast, L-DOPA–treated DM rats had preserved SF thresholds at 6 weeks after STZ, and were decreased by 15.5% relative to controls, changing by less than 1% after the initiation of L-DOPA treatment, and were significantly preserved compared with DM vehicle rats (Holm–Sidak post hoc, *P* < 0.0001). By 10 weeks after STZ, the SF thresholds of DM vehicle rats continued to decrease, showing a 34.5% decrease compared with control vehicle rats (Holm–Sidak post hoc, *P* < 0.0001). DM DOPA rats showed a milder, 24.0% decrease in SF thresholds relative to control vehicle rats (Holm–Sidak post hoc, *P* < 0.0001), but maintained significantly better visual function than DM vehicle rats (Holm–Sidak post hoc, *P* < 0.0001).

### Daily L-DOPA Treatment Recovered Retinal Function in Rod-dominated ERG Responses in Diabetic Rats

**Figure 5.**
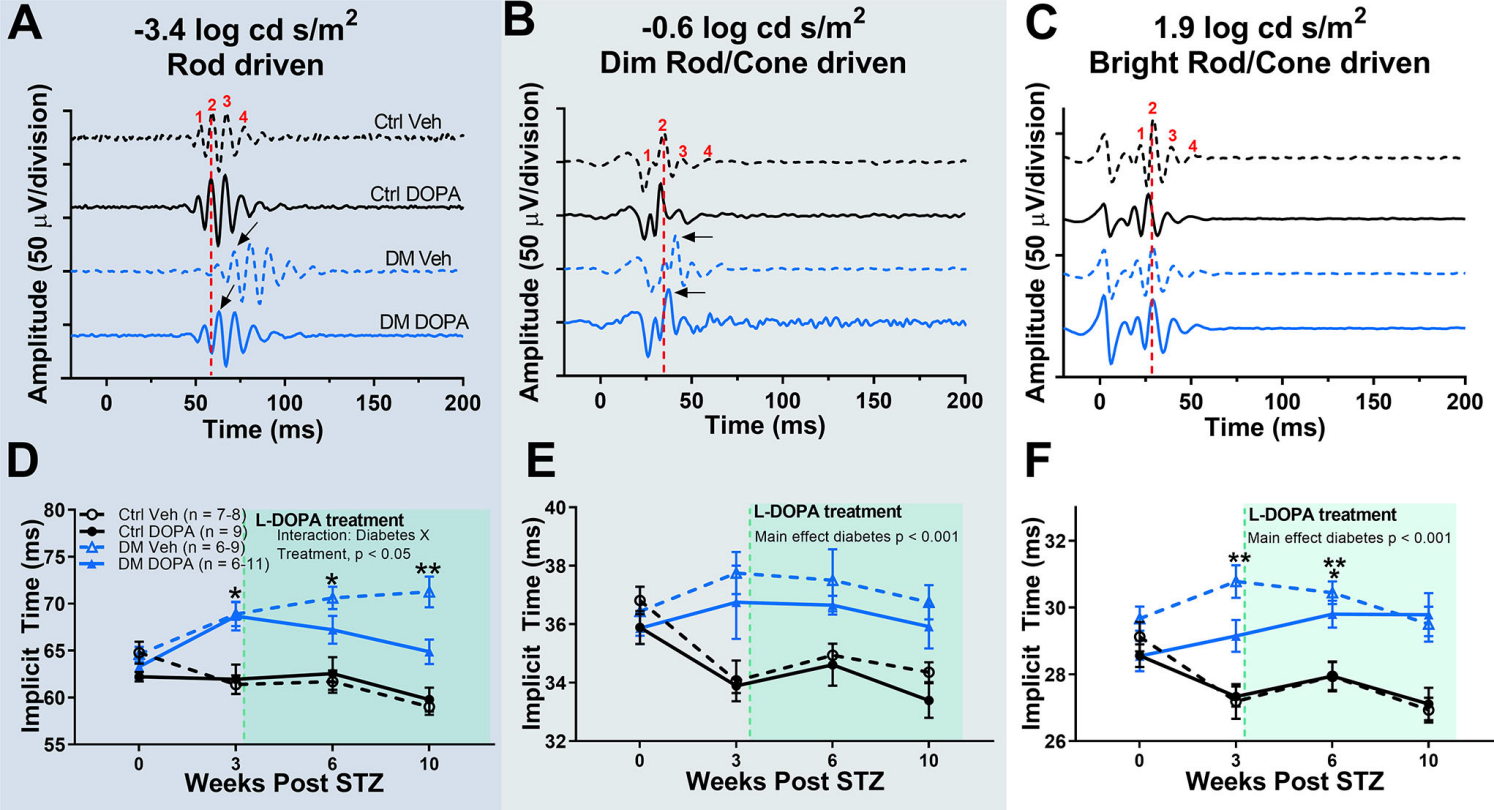
L-DOPA treatment selectively recovers OP2 implicit time for rod-driven ERG responses. (A, B, C) Representative ERG recordings from each treatment group at 10 weeks after STZ for rod-driven and rod/cone-driven responses. (D) Average OP2 implicit times across time in response to rod-driven dim flash stimuli showed significant delays by 3 weeks, which recovered with daily L-DOPA treatment, three-way repeated-measure ANOVA, interaction effect of diabetes and treatment, F (1, 33) = 4.403, *P* = 0.0436. (E, F) OP2 implicit times in response to both dim and bright mixed rod/cone-driven stimuli showed significant delays by 3 weeks which were not altered by daily L-DOPA treatment (three-way repeated-measure ANOVA, main effect diabetes, F (1, 86) = 39.01, *P* < 0.0001 and F (1, 90) = 89.93, *P* < 0.0001, respectively). Data plotted as mean ± standard error of the mean. *P < 0.05; **P < 0.01.

After a 12.6% delay in OP2 implicit time in response to dim stimuli (–3.4 log cd s/m^2^) at 3 weeks after STZ, DM DOPA rats recovered OP2 implicit time at each subsequent measurement after the onset of L-DOPA (Figs. 5A and 5D), three-way repeated-measure ANOVA, F (1, 33) = 4.403, *P* = 0.0436. The OP2 implicit times in response to dim stimuli from DM vehicle rats became increasingly delayed by 4% at 6 weeks after STZ (Holm–Sidak post hoc, *P* = 0.0052), whereas DM DOPA rats recovered OP2 implicit time by 6%. At 10 weeks after STZ, DM vehicle rats continued to show significantly delayed OP2 implicit time in response to –3.4 log cd s/m^2^ responses compared with control vehicle (Holm–Sidak post hoc, *P* = 0.0028), whereas DM DOPA rats had recovered OP2 implicit times that were not significantly delayed compared with control vehicle rats (Holm–Sidak post hoc, *P* = 0.1092).

L-DOPA treatment had no significant effect on OP2 implicit time for mixed rod/cone responses (–0.6 and 1.9 log cd s/m^2^). Regardless of treatment, DM rats showed delayed implicit times at each time point (Figs. 5B–5F; –0.6 log cd s/m^2^: main effect of diabetes, *P* < 0.001; 1.9 log cd s/m^2^: main effect of diabetes, *P* < 0.001). In keeping with previous findings,^17^^,^^27^^,^^30^^,^^38^ OP implicit times generated by rod-dominated stimuli were the most sensitive to diabetic changes.

### Daily L-DOPA Treatment Significantly Increased Retinal L-DOPA Levels and Recovered Retinal Dopamine and DOPAC Levels in Diabetic Rats

At 10 weeks after STZ injection, we confirmed dopamine deficiency in DM rats and assessed the bioavailability of L-DOPA in the retina to confirm that L-DOPA was reaching the retinal tissue after systemic oral dosing. We then assessed whether or not L-DOPA treatment could recover dopamine and DOPAC in the retina.

**Figure 6.**
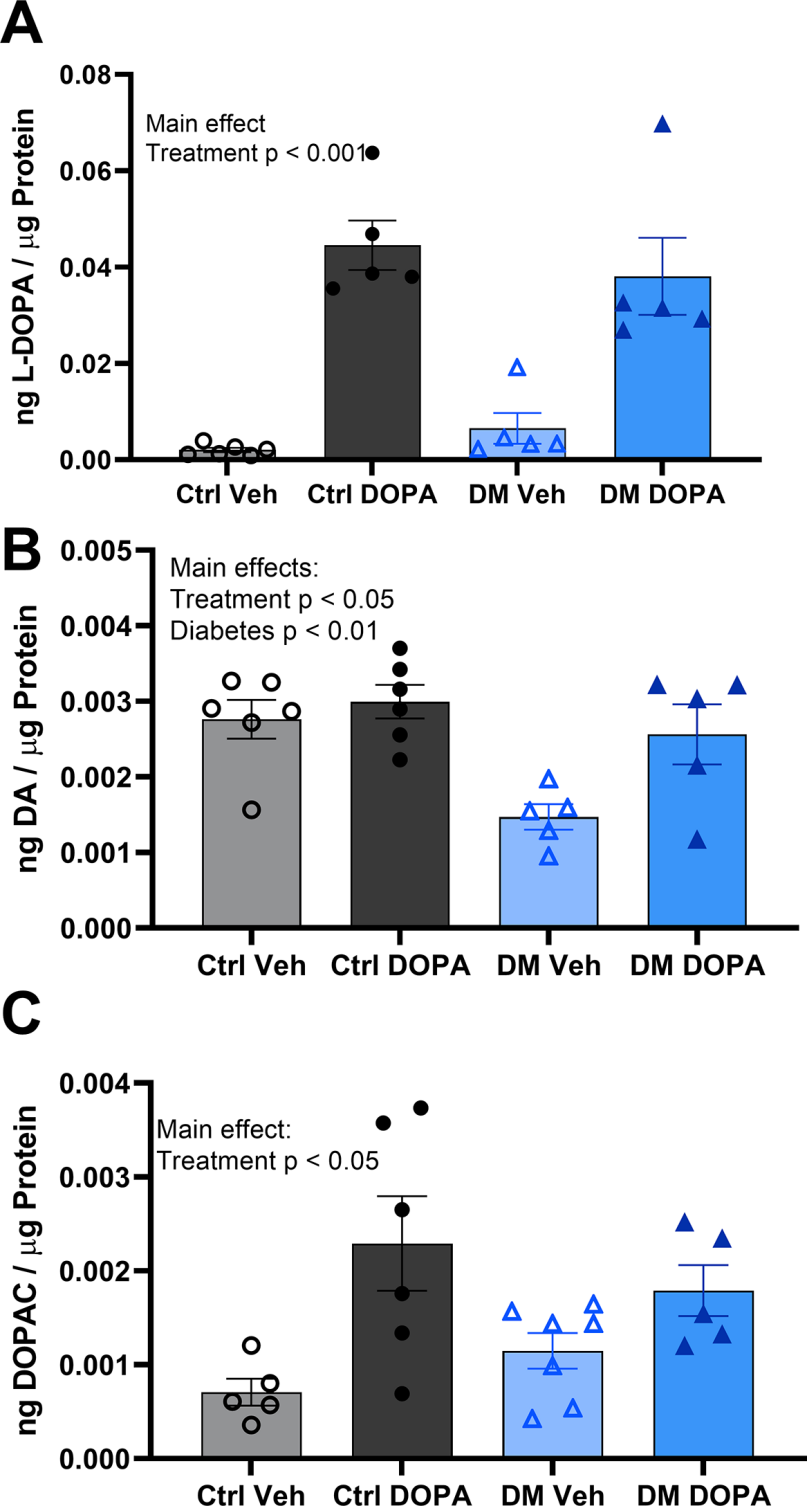
L-DOPA treatment restored reduced retinal dopamine in diabetic rats. L-DOPA treatment significantly increased (A) L-DOPA, (B) dopamine, and (C) DOPAC levels compared with vehicle treated rats, two-way ANOVA, main effects: F (1, 18) = 44.98, *P* < 0.0001; F (1, 18) = 5.960, *P* = 0.0252; and F (1, 17) = 19.06, *P* = 0.0004, respectively. Diabetes significantly decreased (B) dopamine levels in the retina, two-way ANOVA, main effect F (1, 18) = 10.12, *P* = 0.0052), but not L-DOPA or DOPAC levels. Data plotted as mean ± standard error of the mean.

L-DOPA treatment led to a significant, robust increase in retinal L-DOPA content (Fig. 6A), two-way ANOVA, F (1, 18) = 44.98, *P* < 0.0001. The L-DOPA content increased by more than 500% in rats treated with L-DOPA, and diabetes did not significantly affect retinal L-DOPA content. However, similar to previous studies,^17^^,^^18^^,^^30^ diabetes led to a significant decrease in the retinal dopamine content with DM vehicle rats, showing a 47.6% decrease in retinal DA compared with control vehicle rats (Fig. 6B) two-way ANOVA, main effect F (1, 18) = 10.12, *P* = 0.0052. L-DOPA treatment significantly elevated retinal dopamine levels (Fig. 6B), main effect of treatment, F (1, 18) = 5.96, *P* = 0.0252. The dopamine levels in DM DOPA rats were indistinguishable from control vehicle values.

Although diabetes did not significantly affect retinal DOPAC content, L-DOPA treatment led to a significant increase in DOPAC (Fig. 6C), two-way ANOVA, main effect F (1, 22) = 10.01, *P* = 0.0045. DM DOPA rats showed increased DOPAC by 32.4% compared with DM vehicle rats, which was not statistically significant (Holm–Sidak post hoc, *P* = 0.3726). Similarly, control DOPA rats also showed a robust increase in DOPAC content, increasing by 61.2% compared with control vehicle rats (Holm–Sidak post hoc, *P* = 0.0191).

## Discussion

This study showed that L-DOPA treatment is protective against early stage DR when started at the first signs of measurable visual and retinal dysfunction in diabetic rats. These early changes in visual and retinal function measured by OMR decreases and OP delays, respectively, are in agreement with previous work and precede vascular changes such as blood vessel leakage, pericyte dropout, and acellular “ghost” capillaries.^39^^–^^41^

Furthermore, our data reproduce previously reported reductions in retinal dopamine induced by chronic hyperglycemia^17^^,^^30^ and shows that L-DOPA treatment ameliorated dopamine deficiencies in the diabetic retina. Taken together, these results support a clinically feasible approach to treating DR with L-DOPA, starting treatment after patients with diabetes show OP delays or early subtle changes in visual function that precede severe vision loss and current clinical, vascular hallmarks of DR.

### Early Visual and Retinal Dysfunction to Identify an Early Treatment Window for DR

The current study asked the question: Could early changes in retinal and visual function identify a therapeutic window to start L-DOPA treatment that provides the same efficacy as starting treatment at the initiation of hyperglycemia? The role of dopamine in the pathogenesis of DR has been studied extensively,^17^^–^^19^^,^^30^^,^^42^ and L-DOPA has been shown to have a protective role in DR.^5^^,^^17^^,^^30^ Previous preclinical experiments started L-DOPA treatment at the onset diabetes, before any signs of retinal or visual dysfunction.^17^^,^^30^ This is clinically analogous to prescribing a type 1 diabetic patient L-DOPA when they are first diagnosed with type 1 diabetes. Although the prior experiments provide evidence that L-DOPA could be protective against DR, they are limited in providing insight for where L-DOPA treatment could fit in with the current and future clinical DR screening and treatment standards.

The early visual and retinal dysfunction induced by diabetes in this report aligns with other preclinical studies on the STZ rat.^7^^,^^17^^,^^27^^,^^30^^,^^32^^,^^38^ Aung et al.^27^ characterized early visual defects in diabetic rats and also found that significant decreases in visual acuity start at 3 weeks after STZ in similar magnitudes to those reported here. Furthermore, the present study also matched previous studies in demonstrating that rod-driven pathways were the most acutely affected by diabetes and were measurable after 3 to 4 weeks of hyperglycemia.^17^^,^^27^^,^^30^^,^^38^

Although previous studies using animal models of DR showed that L-DOPA prevented delays in OPs and slowed disease progression, we found here that daily L-DOPA treatment started after significant OP delays recovered rod-driven OP implicit times to nondiabetic control values. These data align with a recent study of L-DOPA treatment in patients with diabetes, without retinopathy, where L-DOPA actually recovered the OP implicit time over the 2-week course of treatment for dim, rod-driven ERG responses.^5^ Taken together, these data suggest that retinal neuronal plasticity is present at an early stage of retinopathy, preceding vascular lesions.

Additionally, it is noteworthy that L-DOPA provided benefit to only select ERG waves in this study:, namely, OP and a-wave implicit times in response to dim flash stimuli. These data mirror the clinical data showing L-DOPA treatment benefiting only the OP and a-wave implicit times generated by dim, rod-driven stimuli in diabetic retina and not providing significant benefit to the b-wave response.^5^ Although the underlying mechanisms of this selective effect are unknown, it may be due to the cellular origins of waveform components, differential susceptibility of these cells to dysfunction induced by diabetes, and/or expression of dopamine receptors on different retinal cell types. Additional studies are needed to investigate the mechanisms of L-DOPA's protective effects in DR.

### L-DOPA Treatment Alters Dopaminergic Monoamine Content in the Retina

Previous studies investigating L-DOPA as a treatment for DR have not measured retinal L-DOPA levels. Although it is presumed that L-DOPA passes through the blood–retina barrier and is converted into dopamine in the retina, previous studies do not show that L-DOPA restores retinal dopamine or DOPAC. The effect of L-DOPA treatments on other dopaminergic tissues, such as the striatum and hippocampus, is to increase endogenously produced dopamine in neuronal tissues.^31^^,^^43^^,^^44^ In the rat striatum, L-DOPA reliably increases both dopamine and DOPAC content.^45^^,^^46^ Increased DOPAC is usually attributed to increased dopamine turnover suggesting an increase in dopaminergic activity.^47^ The present study suggests that L-DOPA can exert a desired pharmacologic effect in the retina by increasing retinal dopamine and DOPAC, similar to what is observed in other tissues. Furthermore, this study supports the idea that L-DOPA treatment results in high bioavailability of L-DOPA in the retina and suggests that retinal tissue can store relatively large amounts of the compound.

Dopamine has many different functions in the retina, including the regulation of circadian rhythms and mediating adaptation to different lighting conditions by opening/closing gap function.^48^ Whether or not dopamine preserves SF thresholds by increasing center surround inhibition or by directly preventing damage to the neurovascular complexes that are altered in DM remains unclear.

### Clinical Relevance

The current diagnosis and treatment paradigm for DR hinges on routine, high-throughput screening of patients with diabetes for changes in retinal vasculature via fundus photography.^49^^,^^50^ However, there is increasing evidence that delays in OP latency are detectable before changes in vasculature are revealed on fundus photography.^7^^,^^11^^,^^17^^,^^38^ Advancements in ERG technology are making ERG screening more common. Recent studies report that novel hand-held ERGs are suitable for primary care clinics, can detect vision-threating DR with comparable sensitivity and specificity with fundus photography, and can detect preclinical OP latency delays in patients with diabetes with angiographically normal retinas.^4^^,^^5^^,^^49^^,^^51^

With recent technological advancements to the ERG,^4^^,^^5^^,^^49^^,^^52^ the growing body of evidence backing OP latency as a marker for early DR,^5^^,^^10^^,^^11^^,^^30^^,^^38^^,^^53^ and increasing emphasis on early detection and screening initiatives for patients with diabetes,^50^^,^^51^^,^^54^ ERG has the potential to grow into a major screening method for DR. The current study supports that screening for early stage DR with ERG may be valuable in identifying a new therapeutic window for a new class of dopamine-related treatment strategies for DR. These data suggest that detecting and treating DR at this stage in the disease may slow the progression of retinopathy before vascular pathologies appear and protect from downstream vision loss.

In the current study, L-DOPA treatment recovered retinal dopamine levels in diabetic rats, recovered retinal function, and slowed progression of visual deficits. However, DR is classically diagnosed as a vascular disease of the eye, and it is important in future studies to address whether or not L-DOPA treatment affects the vascular stages of the disease where the vision is most likely to be lost. Importantly, dopamine is a known anti-VEGF compound that acts through the D2 dopamine receptors to induce endocytosis of VEGF receptor 2, which is critical for promoting angiogenesis.^55^^,^^56^ Intravitreal injections of anti-VEGF compounds are currently one of the primary treatment to manage vascular pathology in late-stage DR.^57^^–^^59^ Thus, L-DOPA treatment cannot be ruled out as a potential neuroprotective agent acting on vascular pathology in DR.

Although these data are promising, L-DOPA treatment is not without limitations, especially when prescribed for the long-term management of Parkinson's disease. After 5 years of levodopa therapy, the majority of patients suffer from fluctuations, dyskinesias, toxicity, or loss of efficacy.^60^ These fluctuations can be ameliorated by changing the treatment paradigm to a combination therapy of L-DOPA and a controlled-release variant, or by the addition of a dopamine agonist.^60^ Dyskinesia typically occurs at higher doses of L-DOPA, and the therapeutic dose for Parkinson's disease increases as patients develop tolerance.^31^^,^^61^^,^^62^ Recent studies have suggested modest protection from DR using a systemic, low-dose formulation in diabetic rats, which could mitigate risk for dyskinesia and toxicity.^32^ Topical eye drop formulations of L-DOPA have also been developed, and have been safely used to study myopia in chicks.^63^ Targeted delivery to the eye and retina could also bypass the limitations and concerns of systemic L-DOPA treatment, but whether or not enough L-DOPA can diffuse to the retina remains unanswered and is a topic for future studies.

In conclusion, we have established a clinically feasible and implementable treatment paradigm that relies on early detection of DR with ERG. This study suggests that early changes in the ERG response detect an early stage in the disease when retinal neuronal plasticity is present, and that retinal function deficits can be reversed with L-DOPA treatment. The next step toward clinical translation is to test whether L-DOPA can alter the course of downstream vascular pathologies.

## Supplementary Material

Supplement 1
