# Supplementary material for: Initiation of L-DOPA Treatment After Detection of Diabetes-Induced Retinal Dysfunction Reverses Retinopathy and Provides Neuroprotection in Rats
Source: Transl Vis Sci Technol. 2021 Apr 13;10(4):8. doi: 10.1167/tvst.10.4.8 (PMC8054623; doi:10.1167/tvst.10.4.8)
Supplement: Supplement 1 [file tvst-10-4-8_s001.docx]

Chesler et al., Initiation of L-DOPA treatment after detection of diabetes-induced retinal dysfunction reverses retinopathy and provides neuroprotection in rats

**Supplemental Material**


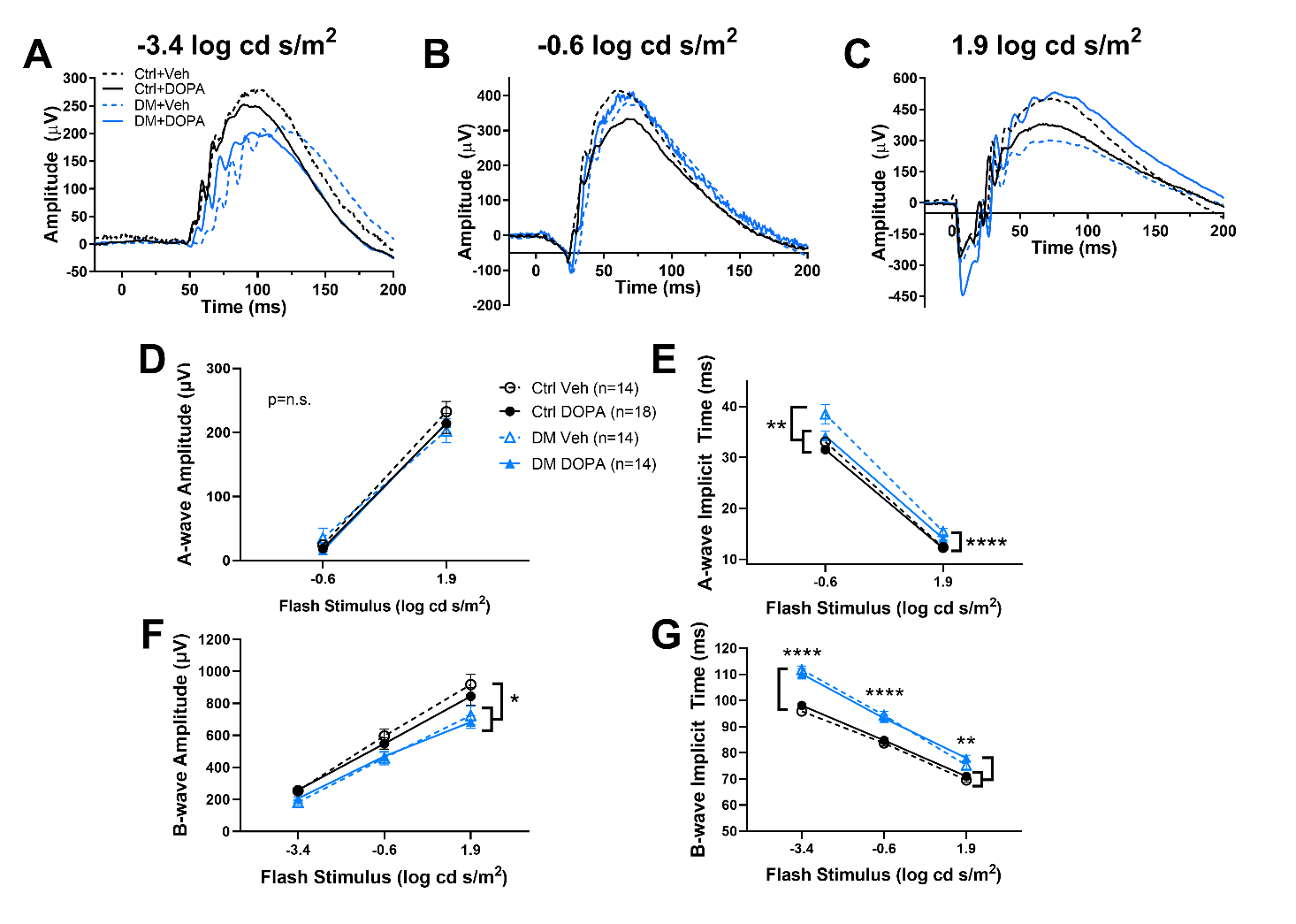


**Supplemental Figure**: L-DOPA treatment provided some benefit to a-wave implicit time in response to dim rod/cone driven stimuli (-0.6 log cd s/m^2^) but no benefit to b-wave amplitude or implicit time at 10 weeks post-STZ. Representative waveforms from each treatment group from rod driven (**A**; -3.4 log cd s/m^2^), dim rod/cone driven (**B**; -0.6 log cd s/m^2^) and bright rod/cone driven (**C**; 1.9 log cd s/m^2^) stimuli. **D**) A-wave amplitude was not different between groups. **E**) A-wave implicit time was significantly different for diabetic vs control groups across time and treatment (Three-way mixed ANOVA for flash*diabetes interaction, F(1,19) = 6.96, p=0.016; Step*treatment interaction, F(1, 19) = 8.48, p=0.009). At -0.6 log cd s/m^2^ stimuli, L-DOPA treatment preserved the a-wave timing, such that only the DM+Veh group was significantly different from all other groups (Tukey’s multiple comparison, p<0.01). However, with the brighter flash stimuli, L-DOPA treatment did not offer protection, with only the DM+Veh group significantly different from the Ctrl groups (Tukey’s multiple comparison, p<0.0001). **F**) B-wave amplitudes were significantly different between diabetic and control groups at the brightest flash stimuli (Three-way mixed ANOVA, flash*diabetes interaction, F(2, 112)=5.86, p=0.004). **G**) B-wave implicit times were significantly different between diabetic and control groups across all flash stimuli (Three-way ANOVA, flash*diabetes interaction, F(2,112) = 25.21, p<0.0001). Symbols represent mean +/- SEM
